# Supplementary material for: Identification of photoperiod-induced specific miRNAs in the adrenal glands of Sunite sheep (Ovis aries)
Source: Front Vet Sci. 2022 Jul 22;9:888207. doi: 10.3389/fvets.2022.888207 (PMC9354845; doi:10.3389/fvets.2022.888207)
Supplement: Supplementary file 1 [file Data_Sheet_1.ZIP › Supplementary file/miRDeep2 file.zip!/miRDeep2 file/Know_Structure/SP42A3/oar-mir-181a-1.pdf]

miRBase precursor	: oar-mir-181a-1
Total read count	: 14267
oar-miR-181a read count	: 14188
remaining reads	: 79

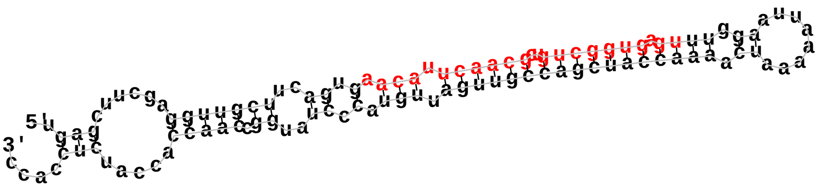

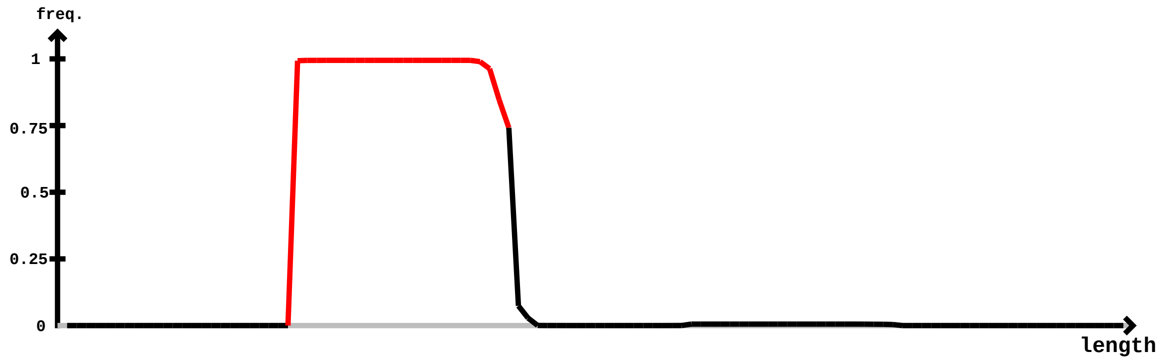


```
                                oar-miR-181a
5'- ugagcuucgagguugcuucagugaacauucaacgcugucggugaguuuggaauuaaaaaucaaaaccaucgaccguugauuguacccuauggccaaccaccaucuccacc   -3'   exp
    .(((......(((((((..((.(.(((.((((((..(((((((.((((.((........)).))))))))))))))))).))).).))..)).))))).....)))....    reads    mm    sample
    .......................aacauucaacgcugucggu....................................................................    69       0     seq
    .......................aacauucaacgcugucggug...................................................................    377      0     seq
    .......................aacauucaacgcugucgguga..................................................................    1673     0     seq
    .......................aacauucaacgcugucggugag.................................................................    1484     0     seq
    .......................aacauucaacgcugucggugagu................................................................    9512     0     seq
    .......................aacauucaacgcugucggugaguu...............................................................    651      0     seq
    .......................aacauucaacgcugucggugaguuu..............................................................    398      0     seq
    .......................aacauucaacgcugucggugaguuug.............................................................    3        0     seq
    .......................aacauucaacgcugucggugaguuugga...........................................................    2        0     seq
    ........................acauucaacgcugucgguga..................................................................    2        0     seq
    ........................acauucaacgcugucggugag.................................................................    1        0     seq
    ........................acauucaacgcugucggugagu................................................................    12       0     seq
    ........................acauucaacgcugucggugaguuu..............................................................    1        0     seq
    .........................cauucaacgcugucggugagu................................................................    1        0     seq
    ..........................auucaacgcugucggugagu................................................................    2        0     seq
    ..............................................................aaaccaucgaccguugauug............................    1        0     seq
    ...............................................................aaccaucgaccguugauuguac.........................    1        0     seq
    ...............................................................aaccaucgaccguugauuguacc........................    1        0     seq
    ................................................................accaucgaccguugaGug............................    1        1     seq
    ................................................................accaucgaccguugauugu...........................    5        0     seq
    ................................................................accaucgaccguugauugua..........................    4        0     seq
    ................................................................accaucgaccguugauuguac.........................    11       0     seq
    ................................................................accaucgaccguugauuguacc........................    55       0     seq
```
